# Supplementary material for: Single-cell transcriptomics of the goldfish retina reveals genetic divergence in the asymmetrically evolved subgenomes after allotetraploidization
Source: Commun Biol. 2022 Dec 26;5:1404. doi: 10.1038/s42003-022-04351-3 (PMC9792465; doi:10.1038/s42003-022-04351-3)
Supplement: Supplementary file 2 — Supplementary Information [file 42003_2022_4351_MOESM2_ESM.pdf]

Single-cell transcriptomics of the goldfish retina reveals genetic divergence in the asymmetrically evolved subgenomes after allotetraploidization

Tetsuo Kon, Kentaro Fukuta, Zelin Chen, Koto Kon-Nanjo, Kota Suzuki, Masakazu Ishikawa, Hikari Tanaka, Shawn M. Burgess, Hideki Noguchi, Atsushi Toyoda, Yoshihiro Omori

Supplementary Figures

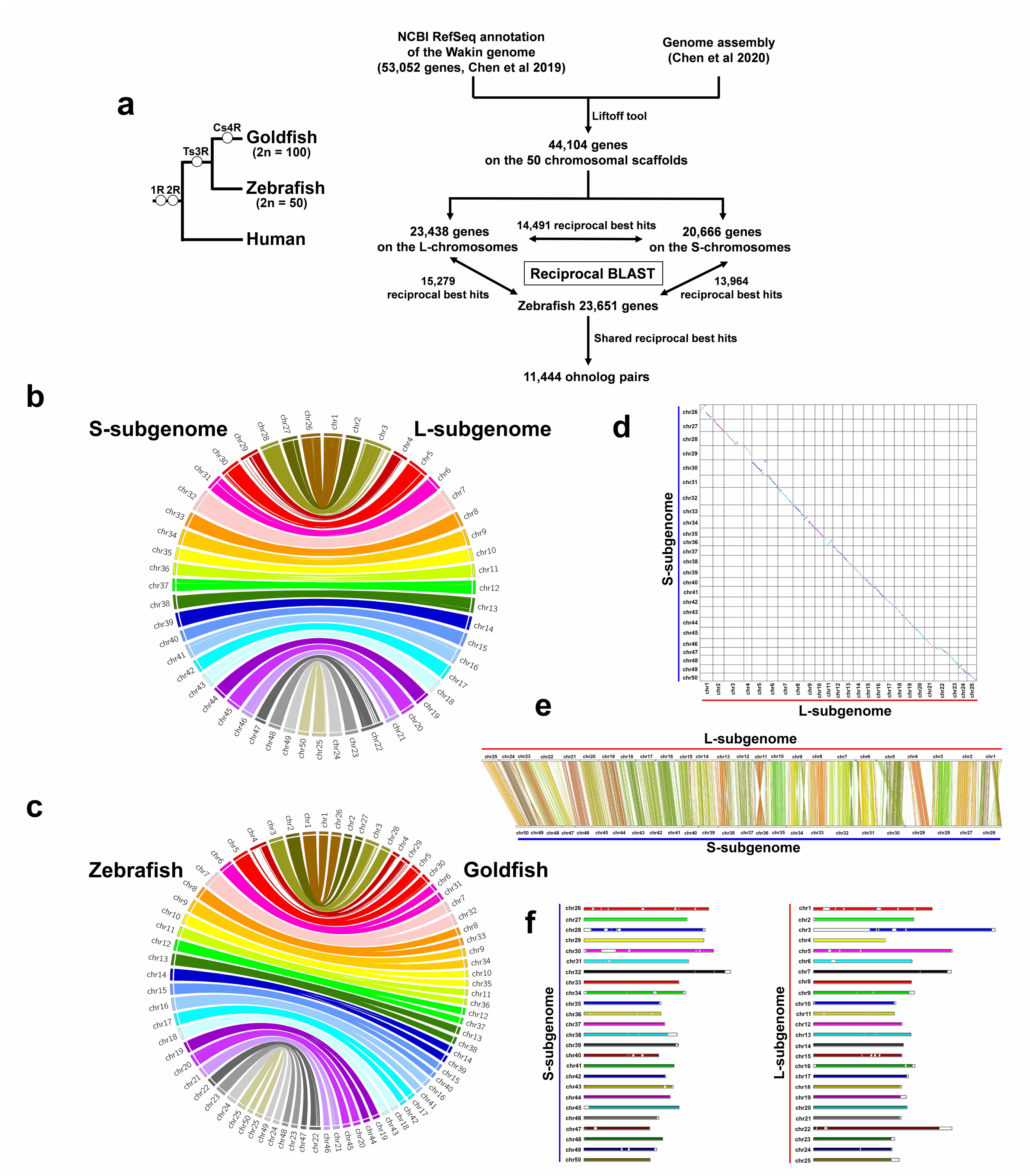

Supplementary Figure 1. Identification of the 11,444 ohnolog pairs in goldfish and zebrafish chromosomes

(a) Left panel; Cladogram showing the evolutionary relationships between goldfish, zebrafish, and humans. The white circles indicate the two rounds of whole genome duplication in the vertebrate ancestor (1R and 2R), Ts3R, and Cs4R. Right panel; Pipeline for identification of 11,444 ohnolog pairs in goldfish genome. (b) Circos plot depicting the 11,444 ohnolog pairs. The L-chromosomes are located on the right and the S-chromosomes are located on the left. (c) Circos plot depicting the ortholog pairs between the 11,444 zebrafish genes and 11,444 goldfish ohnolog pairs. The 50 goldfish chromosomes are located on the right. The 25 zebrafish chromosomes are located on the left. (d) Oxford dot plot of the 11,444 ohnolog pairs. (e) Dual synteny plot of the 11,444 ohnolog pairs. (f) Bar plot showing the chromosomal position of each ohnolog.

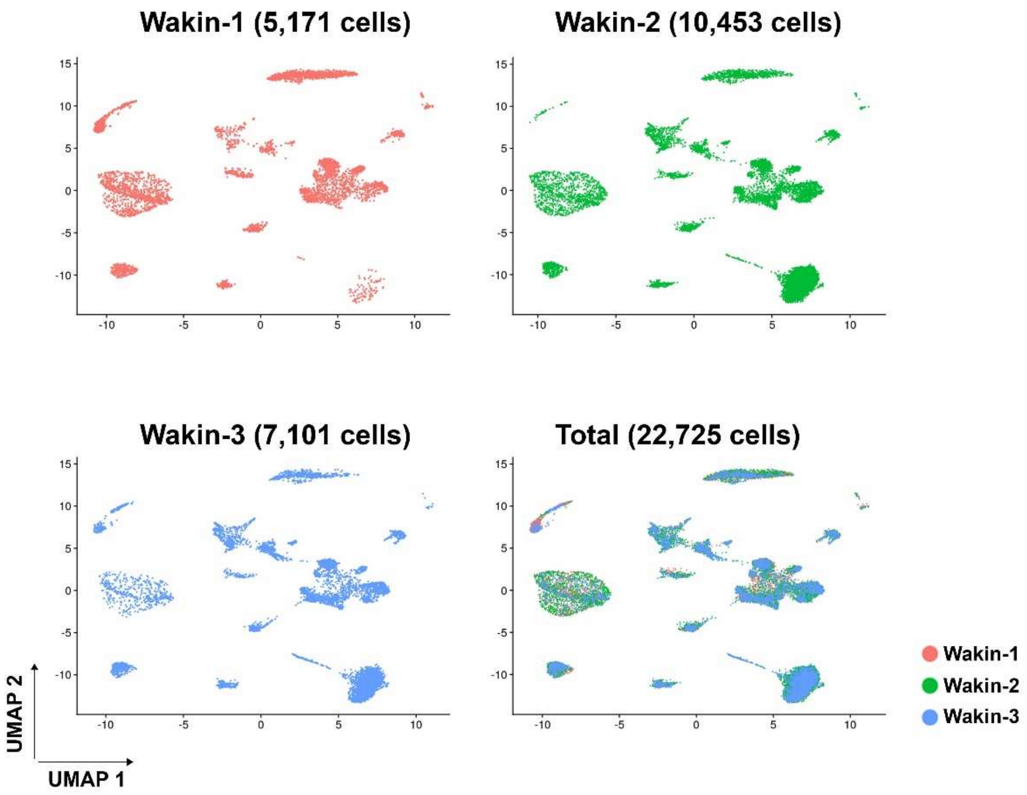

Supplementary Figure 2. UMAP plots of scRNA-seq data for three Wakin goldfish

The scRNA-seq data from three Wakin individuals (red, green, and blue) are shown on the UMAP plot.

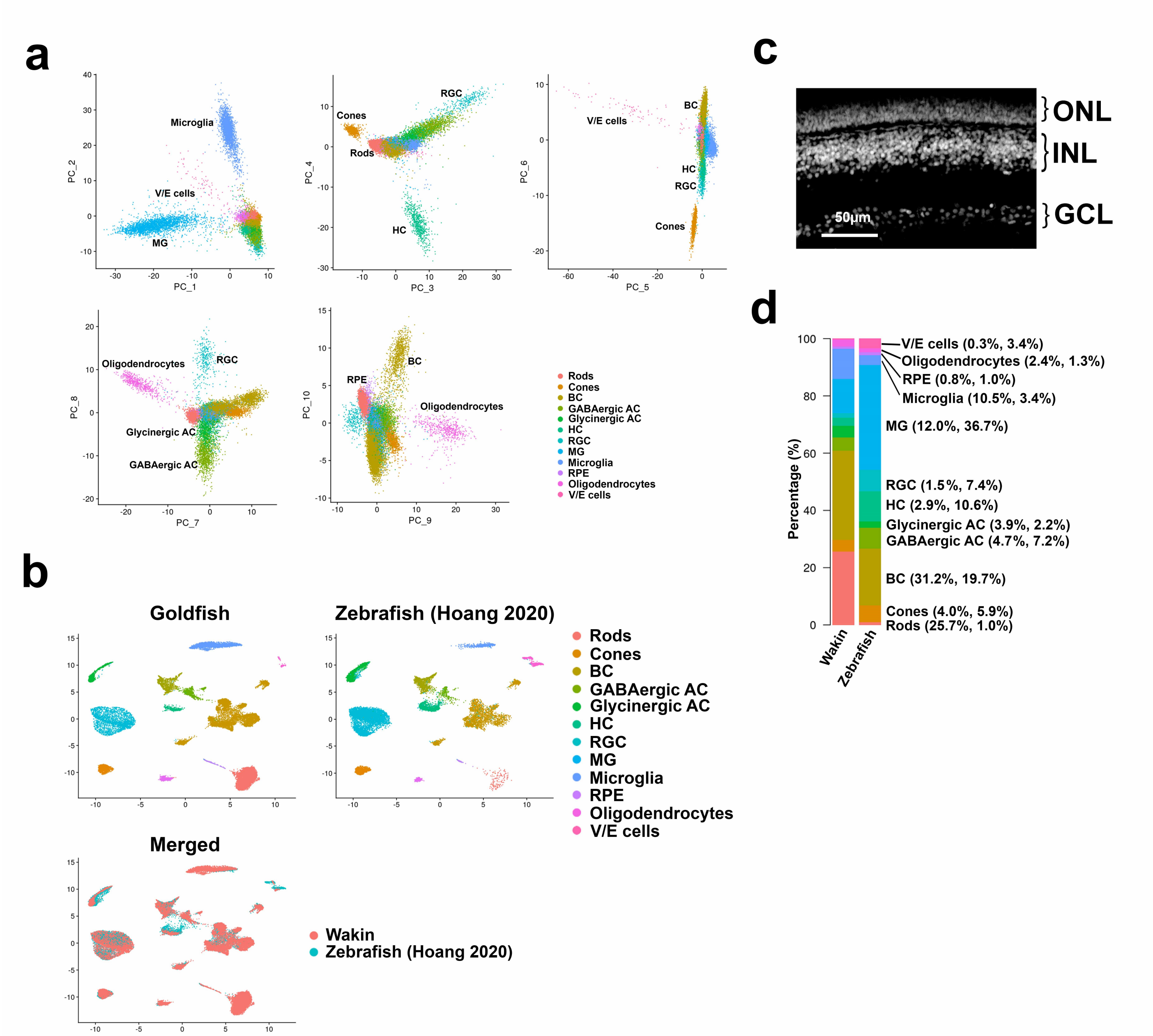

Supplementary Figure 3. Identification of retinal cell types in goldfish

(a) PCA plots of the first 10 components of scRNA-seq data from Wakin goldfish retina. The 12 retinal cell types formed distinct clusters. (b) UMAP plots showing goldfish and zebrafish scRNA-seq data. The cell clusters were assigned to 12 retinal cell types in goldfish or zebrafish. (c) DAPI staining of goldfish retinal sections. The outer nuclear layer (ONL), the inner nuclear layer (INL), and the ganglion cell layer (GCL) are shown. (d) Percentage of cell types identified by scRNA-seq analysis in goldfish and zebrafish. Twelve cell types identified were shown. Abbreviations: V/E cells, vascular endothelial cells; RPE, retinal pigmented epithelial cells; MG, Müller glia; RGC, retinal ganglion cells; HC, horizontal cells; AC, amacrine cells; BC, bipolar cells.

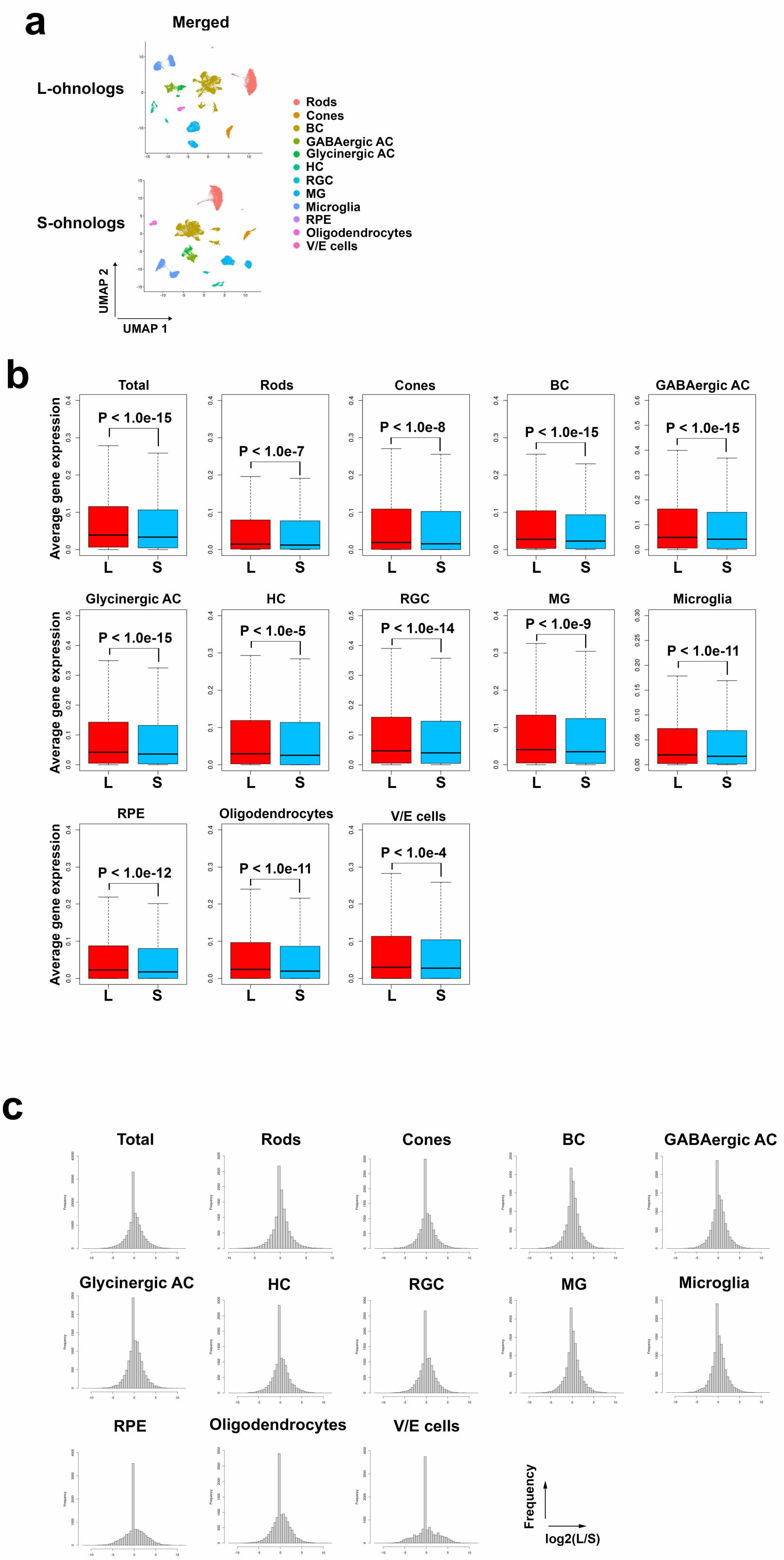
a
Merged
L-ohnologs
S-ohnologs
UMAP 2
UMAP 1
Rods
Cones
BC
GABAergic AC
Glycinergic AC
HC
RGC
MG
Microglia
RPE
Oligodendrocytes
V/E cells
b
Average gene expression
Total
P < 1.0e-15
Rods
P < 1.0e-7
Cones
P < 1.0e-8
BC
P < 1.0e-15
GABAergic AC
P < 1.0e-15
Glycinergic AC
P < 1.0e-15
HC
P < 1.0e-5
RGC
P < 1.0e-14
MG
P < 1.0e-9
Microglia
P < 1.0e-11
RPE
P < 1.0e-12
Oligodendrocytes
P < 1.0e-11
V/E cells
P < 1.0e-4
L
S
c
Total
Rods
Cones
BC
GABAergic AC
Glycinergic AC
HC
RGC
MG
Microglia
RPE
Oligodendrocytes
V/E cells
Frequency
log2(L/S)

Supplementary Figure 4. Gene expression level of L- and S-ohnologs in the 12 retinal cell types

(a) UMAP plots based on the expression data of the 11,444 L-ohnologs (upper panel) and the 11,444 S-ohnologs (lower panel) were shown independently. The 12 cell types identified were shown in different colors. (b) The distribution of the averaged gene expression levels of L-ohnologs (red) and S-ohnologs (blue) in the 12 types of retinal cells was shown. The ends of the box are the 25% and 75% quantiles. The horizontal line in the box indicates the median. The lines extending from the top and bottom of the box represent the minimum and maximum values. (c) Histogram of log2 transformed L/S ratios. The L/S peak is centered at zero, suggesting that the most ohnolog pairs show no biased expression. This contrasts with the right-shifted peak observed in Fig. 2c (L/S of total gene expression).

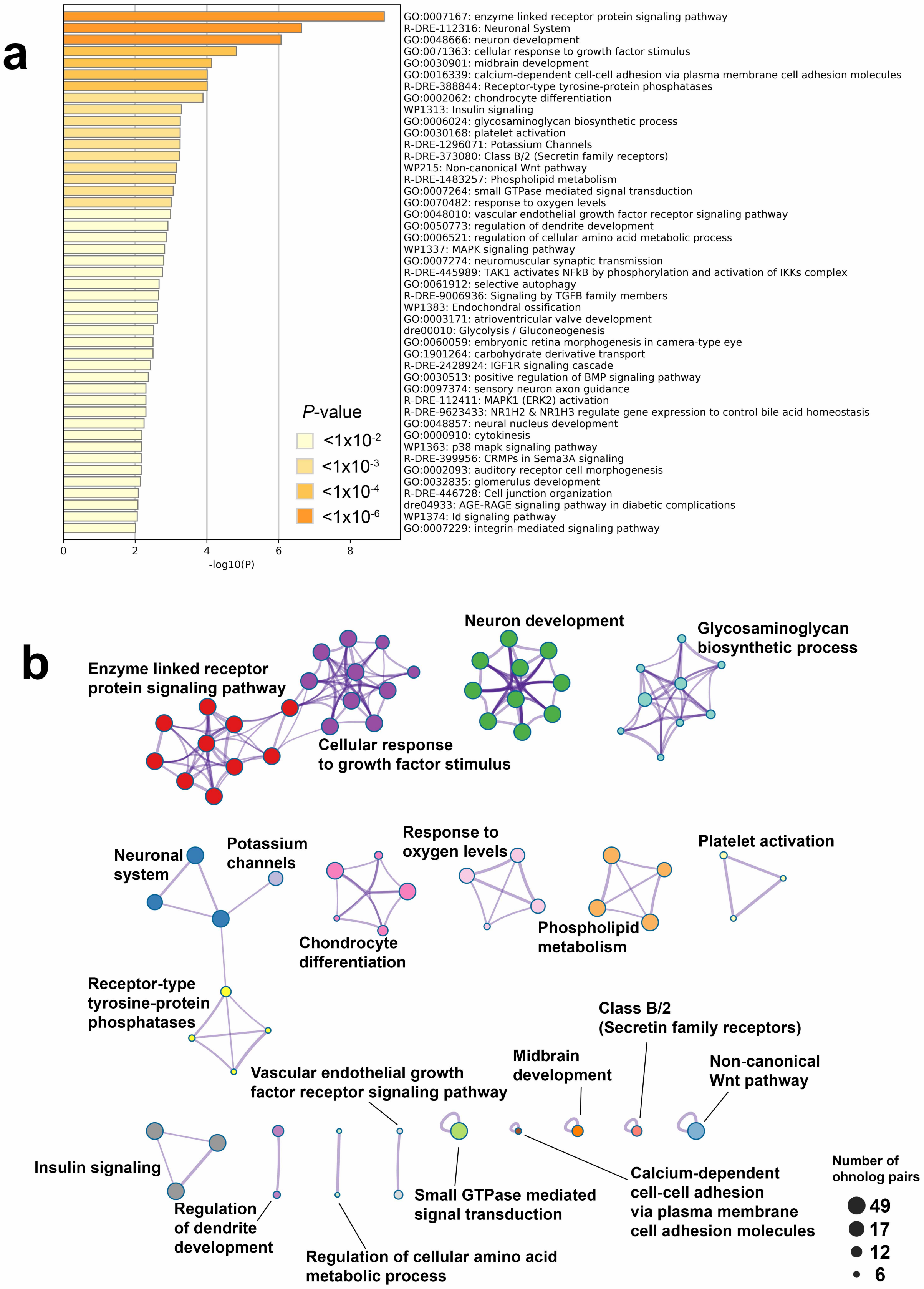
a
GO:0007167: enzyme linked receptor protein signaling pathway
R-DRE-112316: Neuronal System
GO:0048666: neuron development
GO:0071363: cellular response to growth factor stimulus
GO:0030901: midbrain development
GO:0016339: calcium-dependent cell-cell adhesion via plasma membrane cell adhesion molecules
R-DRE-388844: Receptor-type tyrosine-protein phosphatases
GO:0002062: chondrocyte differentiation
WP1313: Insulin signaling
GO:0006024: glycosaminoglycan biosynthetic process
GO:0030168: platelet activation
R-DRE-1296071: Potassium Channels
R-DRE-373080: Class B/2 (Secretin family receptors)
WP215: Non-canonical Wnt pathway
R-DRE-1483257: Phospholipid metabolism
GO:0007264: small GTPase mediated signal transduction
GO:0070482: response to oxygen levels
GO:0048010: vascular endothelial growth factor receptor signaling pathway
GO:0050773: regulation of dendrite development
GO:0006521: regulation of cellular amino acid metabolic process
WP1337: MAPK signaling pathway
GO:0007274: neuromuscular synaptic transmission
R-DRE-445989: TAK1 activates NFkB by phosphorylation and activation of IKKs complex
GO:0061912: selective autophagy
R-DRE-9006936: Signaling by TGFB family members
WP1383: Endochondral ossification
GO:0003171: atrioventricular valve development
dre00010: Glycolysis / Gluconeogenesis
GO:0060059: embryonic retina morphogenesis in camera-type eye
GO:1901264: carbohydrate derivative transport
R-DRE-2428924: IGF1R signaling cascade
GO:0030513: positive regulation of BMP signaling pathway
GO:0097374: sensory neuron axon guidance
R-DRE-112411: MAPK1 (ERK2) activation
R-DRE-9623433: NR1H2 & NR1H3 regulate gene expression to control bile acid homeostasis
GO:0048857: neural nucleus development
GO:0000910: cytokinesis
WP1363: p38 mapk signaling pathway
R-DRE-399956: CRMPs in Sema3A signaling
GO:0002093: auditory receptor cell morphogenesis
GO:0032835: glomerulus development
R-DRE-446728: Cell junction organization
dre04933: AGE-RAGE signaling pathway in diabetic complications
WP1374: Id signaling pathway
GO:0007229: integrin-mediated signaling pathway
P-value
<1x10-2
<1x10-3
<1x10-4
<1x10-6
0
2
4
6
8
-log10(P)
b
Enzyme linked receptor protein signaling pathway
Cellular response to growth factor stimulus
Neuron development
Glycosaminoglycan biosynthetic process
Neuronal system
Potassium channels
Response to oxygen levels
Platelet activation
Chondrocyte differentiation
Phospholipid metabolism
Receptor-type tyrosine-protein phosphatases
Class B/2 (Secretin family receptors)
Midbrain development
Non-canonical Wnt pathway
Vascular endothelial growth factor receptor signaling pathway
Insulin signaling
Small GTPase mediated signal transduction
Calcium-dependent cell-cell adhesion via plasma membrane cell adhesion molecules
Regulation of dendrite development
Regulation of cellular amino acid metabolic process
Number of ohnolog pairs
49
17
12
6

Supplementary Figure 5. Functional enrichment analysis of the 2,430 ohnolog pairs with higher gene expression in the L-ohnolog than in the S-ohnolog

(a) The x-axis represents the negative log10-transformed *P*-value based on the accumulative hypergeometric distribution. The y-axis represents the enriched biological categories. Deeper color of the bar plot means smaller *P*-value. (b) Network representation of the statistically enriched categories in the functional enrichment analysis of the ohnolog pairs with higher gene expression in the L-ohnolog than in the S-ohnolog. The nodes represent the enriched categories and the edges are defined based on the similarities among their gene memberships. The name of the cluster is adopted from the name of the cluster with the smallest *P*-value among the biological categories contained in that cluster. The node size is proportional to the number of input ohnolog pairs grouped into each category.

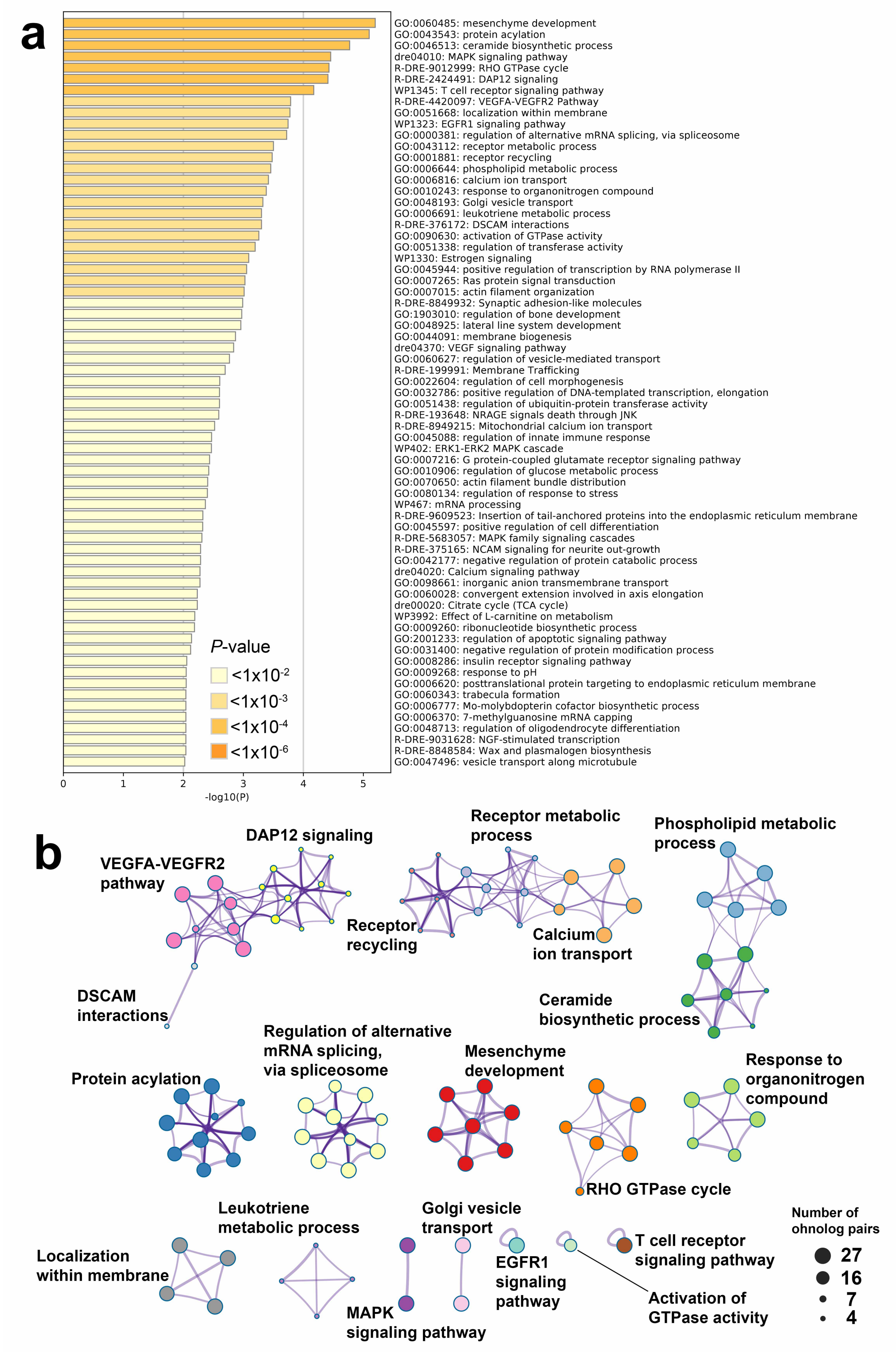
a
GO:0060485: mesenchyme development
GO:0043543: protein acylation
GO:0046513: ceramide biosynthetic process
dre04010: MAPK signaling pathway
R-DRE-9012999: RHO GTPase cycle
R-DRE-2424491: DAP12 signaling
WP1345: T cell receptor signaling pathway
R-DRE-4420097: VEGFA-VEGFR2 Pathway
GO:0051668: localization within membrane
WP1323: EGFR1 signaling pathway
GO:0000381: regulation of alternative mRNA splicing, via spliceosome
GO:0043112: receptor metabolic process
GO:0001881: receptor recycling
GO:0006644: phospholipid metabolic process
GO:0006816: calcium ion transport
GO:0010243: response to organonitrogen compound
GO:0048193: Golgi vesicle transport
GO:0006691: leukotriene metabolic process
R-DRE-376172: DSCAM interactions
GO:0090630: activation of GTPase activity
GO:0051338: regulation of transferase activity
WP1330: Estrogen signaling
GO:0045944: positive regulation of transcription by RNA polymerase II
GO:0007265: Ras protein signal transduction
GO:0007015: actin filament organization
R-DRE-8849932: Synaptic adhesion-like molecules
GO:1903010: regulation of bone development
GO:0048925: lateral line system development
GO:0044091: membrane biogenesis
dre04370: VEGF signaling pathway
GO:0060627: regulation of vesicle-mediated transport
R-DRE-199991: Membrane Trafficking
GO:0022604: regulation of cell morphogenesis
GO:0032786: positive regulation of DNA-templated transcription, elongation
GO:0051438: regulation of ubiquitin-protein transferase activity
R-DRE-193648: NRAGE signals death through JNK
R-DRE-8949215: Mitochondrial calcium ion transport
GO:0045088: regulation of innate immune response
WP402: ERK1-ERK2 MAPK cascade
GO:0007216: G protein-coupled glutamate receptor signaling pathway
GO:0010906: regulation of glucose metabolic process
GO:0070650: actin filament bundle distribution
GO:0080134: regulation of response to stress
WP467: mRNA processing
R-DRE-9609523: Insertion of tail-anchored proteins into the endoplasmic reticulum membrane
GO:0045597: positive regulation of cell differentiation
R-DRE-5683057: MAPK family signaling cascades
R-DRE-375165: NCAM signaling for neurite out-growth
GO:0042177: negative regulation of protein catabolic process
dre04020: Calcium signaling pathway
GO:0098661: inorganic anion transmembrane transport
GO:0060028: convergent extension involved in axis elongation
dre00020: Citrate cycle (TCA cycle)
WP3992: Effect of L-carnitine on metabolism
GO:0009260: ribonucleotide biosynthetic process
GO:2001233: regulation of apoptotic signaling pathway
GO:0031400: negative regulation of protein modification process
GO:0008286: insulin receptor signaling pathway
GO:0009268: response to pH
GO:0006620: posttranslational protein targeting to endoplasmic reticulum membrane
GO:0060343: trabecula formation
GO:0006777: Mo-molybdopterin cofactor biosynthetic process
GO:0006370: 7-methylguanosine mRNA capping
GO:0048713: regulation of oligodendrocyte differentiation
R-DRE-9031628: NGF-stimulated transcription
R-DRE-8848584: Wax and plasmalogen biosynthesis
GO:0047496: vesicle transport along microtubule
P-value
<1x10-2
<1x10-3
<1x10-4
<1x10-6
0 1 2 3 4 5
-log10(P)
b
VEGFA-VEGFR2 pathway
DAP12 signaling
Receptor metabolic process
Phospholipid metabolic process
Receptor recycling
Calcium ion transport
DSCAM interactions
Ceramide biosynthetic process
Regulation of alternative mRNA splicing, via spliceosome
Mesenchyme development
Response to organonitrogen compound
Protein acylation
RHO GTPase cycle
Leukotriene metabolic process
Golgi vesicle transport
Localization within membrane
EGFR1 signaling pathway
T cell receptor signaling pathway
Activation of GTPase activity
MAPK signaling pathway
Number of ohnolog pairs
27
16
7
4

Supplementary Figure 6. Functional enrichment analysis of the 2,188 ohnolog pairs with higher gene expression in the S-ohnolog than in the L-ohnolog

(a) The x-axis represents the negative log10-transformed *P*-value based on the accumulative hypergeometric distribution. The y-axis represents the enriched biological categories. Deeper color of the bar plot means smaller *P*-value. (b) Network representation of the statistically enriched categories in the functional enrichment analysis of the ohnolog pairs with higher gene expression in the S-ohnolog than in the L-ohnolog. The nodes represent the enriched categories and the edges are defined based on the similarities among their gene memberships. The name of the cluster is adopted from the name of the cluster with the smallest *P*-value among the biological categories contained in that cluster. The node size is proportional to the number of input ohnolog pairs grouped into each category.

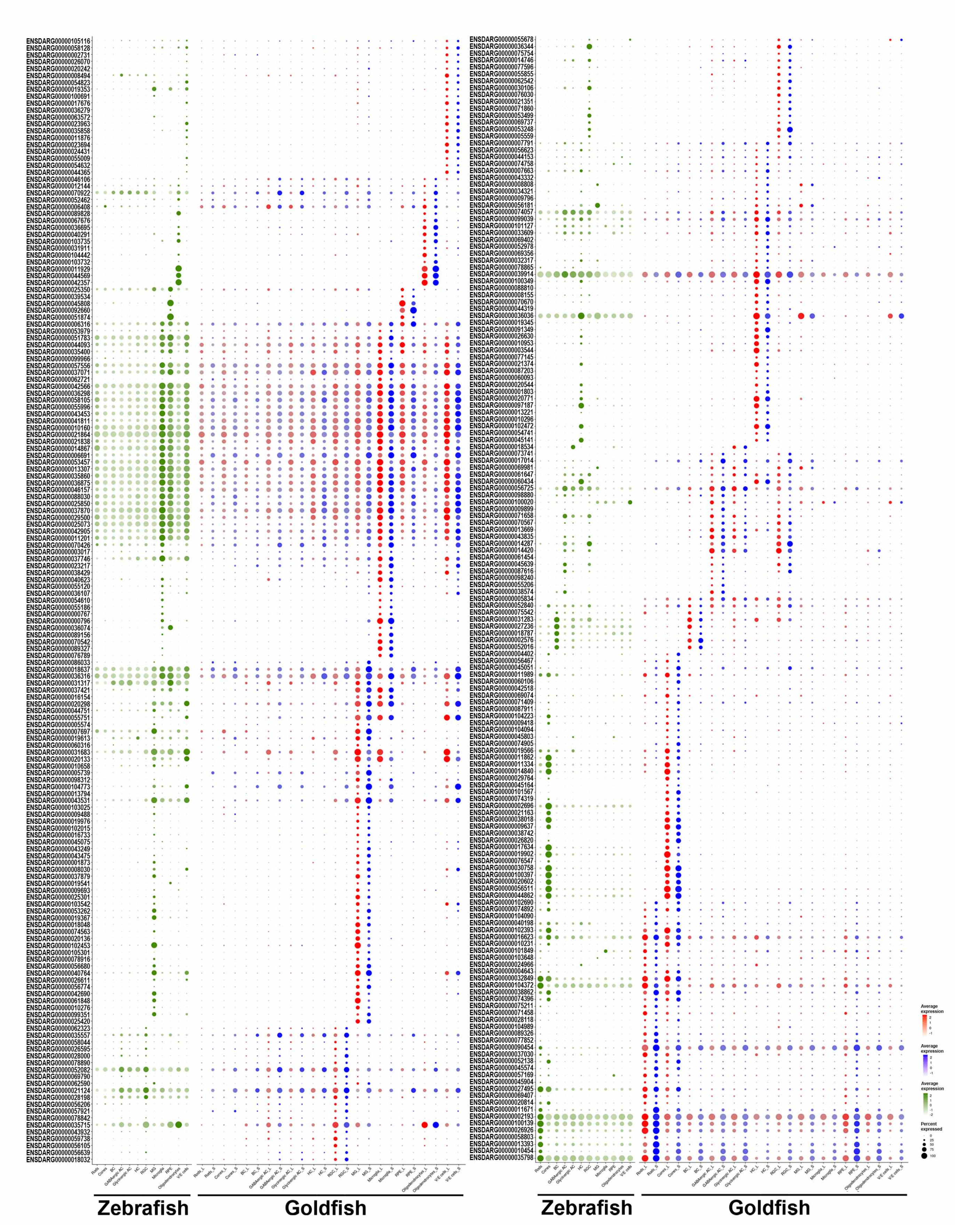
Zebrafish
Goldfish
Zebrafish
Goldfish

Supplementary Figure 7. Gene expression profiles of ohnolog pairs with a similar expression pattern

Gene expression profile of the 326 ohnolog pairs in which both the L-ohnologs (red circles) and S-ohnologs (blue circles) exhibited the similar expression patterns. The expression patterns of zebrafish orthologs are also shown in green circles.

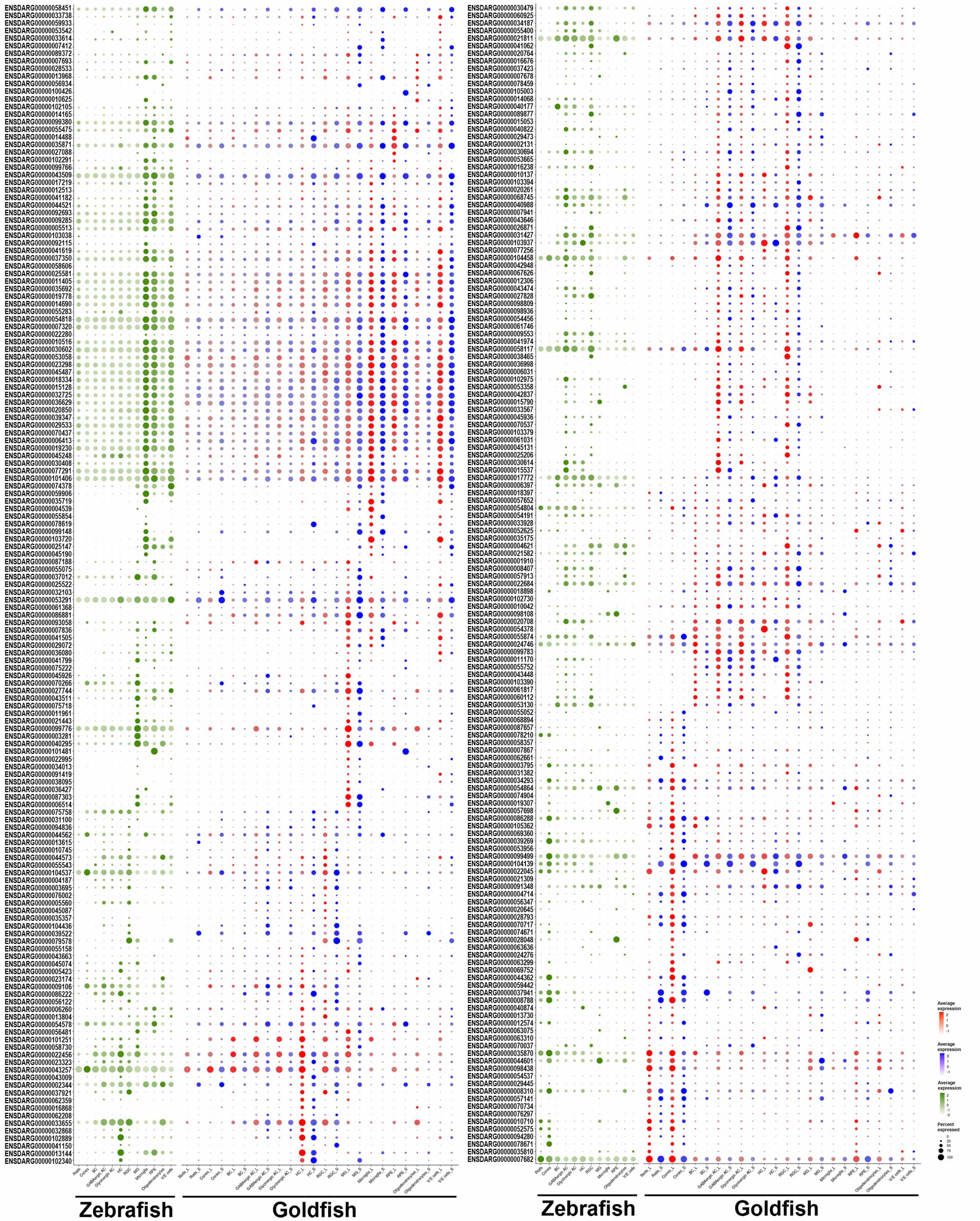
Zebrafish
Goldfish
Zebrafish
Goldfish
Average expression
Average expression
Average expression
Percent expressed

Supplementary Figure 8. Gene expression profiles of ohnolog pairs with a diversified expression pattern

Gene expression profile of the 306 ohnolog pairs showed different expression patterns in the L-ohnologs (red circles) and S-ohnologs (blue circles). The expression patterns of zebrafish orthologs are also shown in green circles.

a

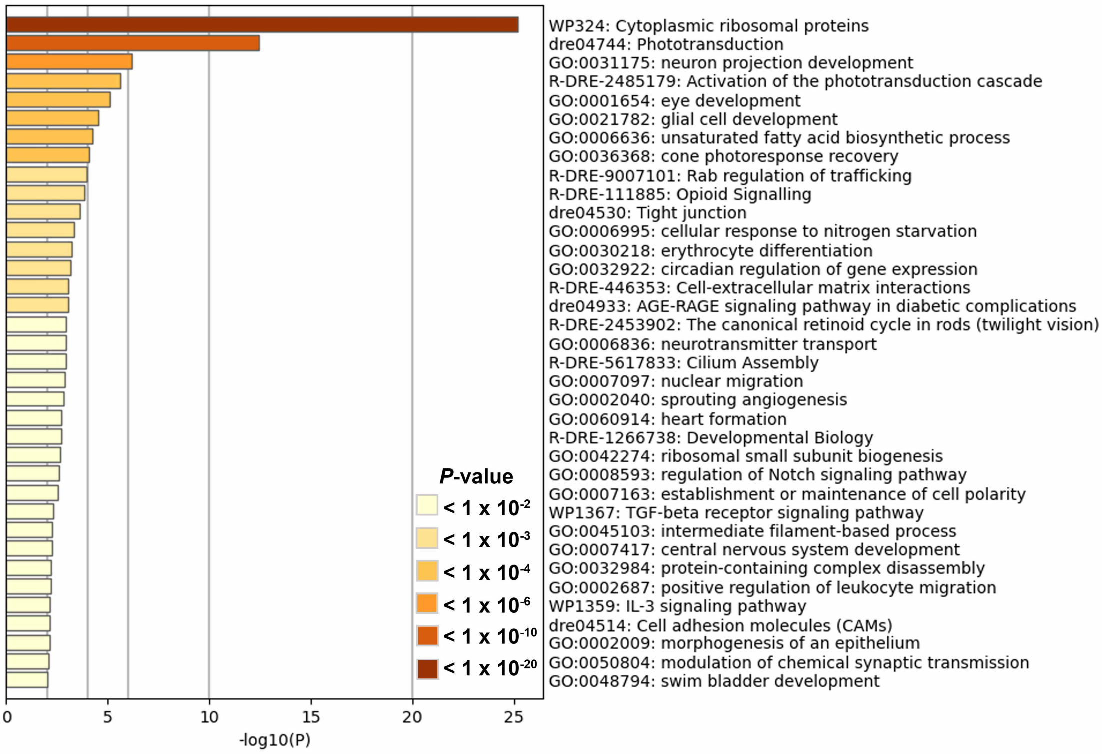
WP324: Cytoplasmic ribosomal proteins
dre04744: Phototransduction
GO:0031175: neuron projection development
R-DRE-2485179: Activation of the phototransduction cascade
GO:0001654: eye development
GO:0021782: glial cell development
GO:0006636: unsaturated fatty acid biosynthetic process
GO:0036368: cone photoresponse recovery
R-DRE-9007101: Rab regulation of trafficking
R-DRE-111885: Opioid Signalling
dre04530: Tight junction
GO:0006995: cellular response to nitrogen starvation
GO:0030218: erythrocyte differentiation
GO:0032922: circadian regulation of gene expression
R-DRE-446353: Cell-extracellular matrix interactions
dre04933: AGE-RAGE signaling pathway in diabetic complications
R-DRE-2453902: The canonical retinoid cycle in rods (twilight vision)
GO:0006836: neurotransmitter transport
R-DRE-5617833: Cilium Assembly
GO:0007097: nuclear migration
GO:0002040: sprouting angiogenesis
GO:0060914: heart formation
R-DRE-1266738: Developmental Biology
GO:0042274: ribosomal small subunit biogenesis
GO:0008593: regulation of Notch signaling pathway
GO:0007163: establishment or maintenance of cell polarity
WP1367: TGF-beta receptor signaling pathway
GO:0045103: intermediate filament-based process
GO:0007417: central nervous system development
GO:0032984: protein-containing complex disassembly
GO:0002687: positive regulation of leukocyte migration
WP1359: IL-3 signaling pathway
dre04514: Cell adhesion molecules (CAMs)
GO:0002009: morphogenesis of an epithelium
GO:0050804: modulation of chemical synaptic transmission
GO:0048794: swim bladder development
P-value
< 1 x 10-2
< 1 x 10-3
< 1 x 10-4
< 1 x 10-6
< 1 x 10-10
< 1 x 10-20
0
5
10
15
20
25
-log10(P)

b

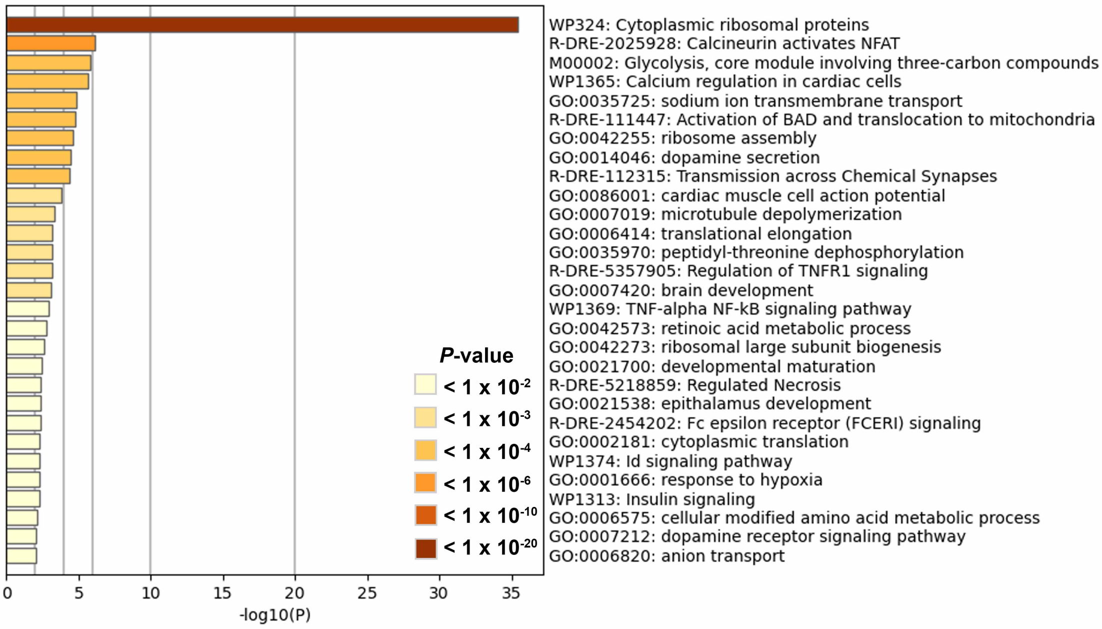
WP324: Cytoplasmic ribosomal proteins
R-DRE-2025928: Calcineurin activates NFAT
M00002: Glycolysis, core module involving three-carbon compounds
WP1365: Calcium regulation in cardiac cells
GO:0035725: sodium ion transmembrane transport
R-DRE-111447: Activation of BAD and translocation to mitochondria
GO:0042255: ribosome assembly
GO:0014046: dopamine secretion
R-DRE-112315: Transmission across Chemical Synapses
GO:0086001: cardiac muscle cell action potential
GO:0007019: microtubule depolymerization
GO:0006414: translational elongation
GO:0035970: peptidyl-threonine dephosphorylation
R-DRE-5357905: Regulation of TNFR1 signaling
GO:0007420: brain development
WP1369: TNF-alpha NF-kB signaling pathway
GO:0042573: retinoic acid metabolic process
GO:0042273: ribosomal large subunit biogenesis
GO:0021700: developmental maturation
R-DRE-5218859: Regulated Necrosis
GO:0021538: epithalamus development
R-DRE-2454202: Fc epsilon receptor (FCERI) signaling
GO:0002181: cytoplasmic translation
WP1374: Id signaling pathway
GO:0001666: response to hypoxia
WP1313: Insulin signaling
GO:0006575: cellular modified amino acid metabolic process
GO:0007212: dopamine receptor signaling pathway
GO:0006820: anion transport
P-value
< 1 x 10-2
< 1 x 10-3
< 1 x 10-4
< 1 x 10-6
< 1 x 10-10
< 1 x 10-20
0
5
10
15
20
25
30
35
-log10(P)

Supplementary Figure 9. Functional enrichment analysis of ohnolog pairs with a diversified expression pattern
(a) Functional enrichment analysis of the 326 ohnolog pairs in which both the L- and S-ohnologs exhibited the similar expression patterns. (b) Functional enrichment analysis of the 306 ohnolog pairs showed different expression patterns in the L- and S-ohnologs. The x-axis represents the negative log10-transformed *P*-value based on the accumulative hypergeometric distribution. The y-axis represents the enriched biological categories.

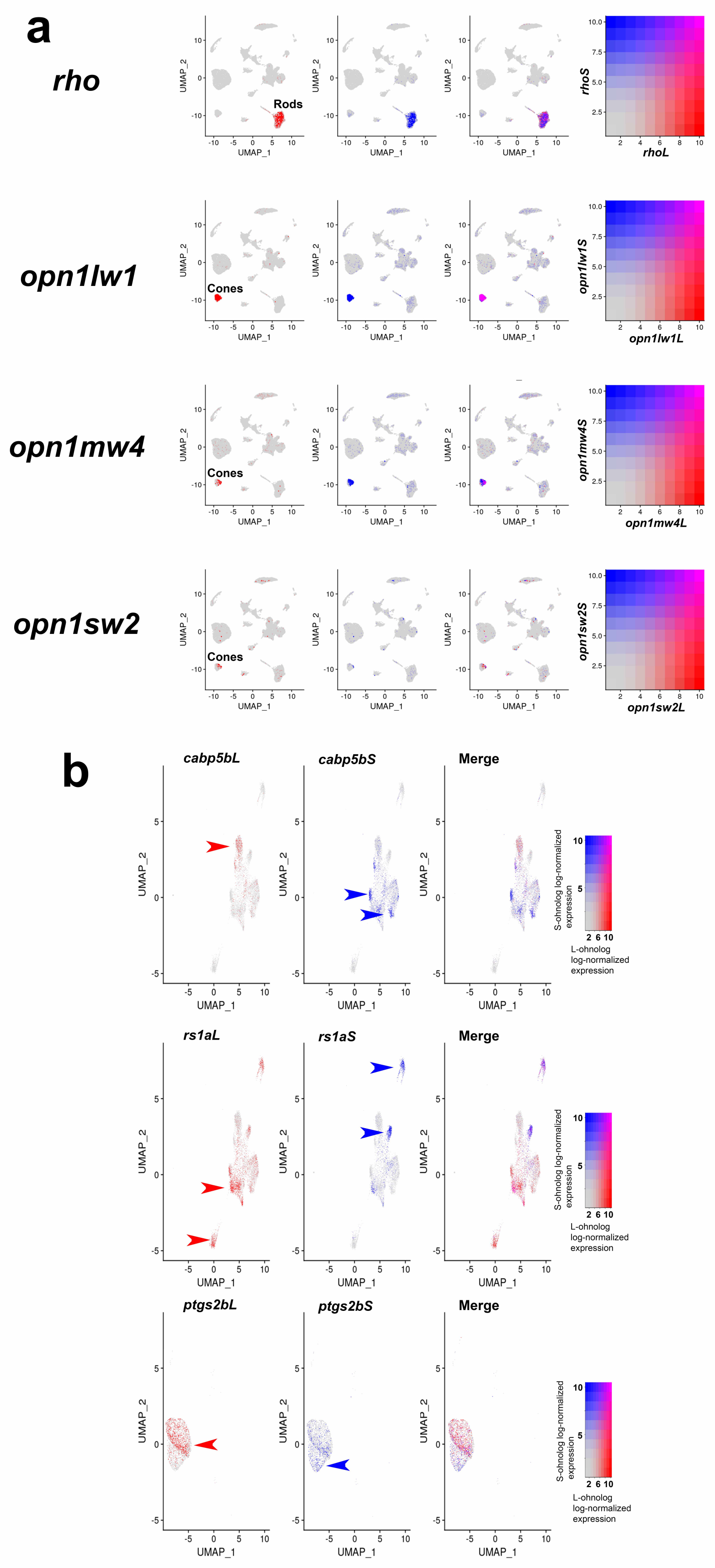
a
rho
Rods
rhoS
rhoL
opn1lw1
Cones
opn1lw1S
opn1lw1L
opn1mw4
Cones
opn1mw4S
opn1mw4L
opn1sw2
Cones
opn1sw2S
opn1sw2L
UMAP_1
UMAP_2
b
cabp5bL
cabp5bS
Merge
rs1aL
rs1aS
Merge
ptgs2bL
ptgs2bS
Merge
S-ohnolog log-normalized expression
L-ohnolog log-normalized expression

Supplementary Figure 10. Expression of ohnolog pairs in the retina. (a) Expression of opsin L/S ohnolog pairs. The gene expression levels of opsin ohnologs in the goldfish retina are shown. (b) Ohnolog pairs in which the L-ohnolog and S-ohnolog show different expression profiles in retinal cell clusters. The *cabp5b* and *rs1a* ohnologs exhibit diversified expression patterns in the bipolar cells of the UMAP cluster. The *ptgs2b* ohnologs exhibit diversified expression pattern in the Müller glia of the UMAP cluster. The red or blue arrowheads show the enriched cells expressing L- or S-ohnologs, respectively.

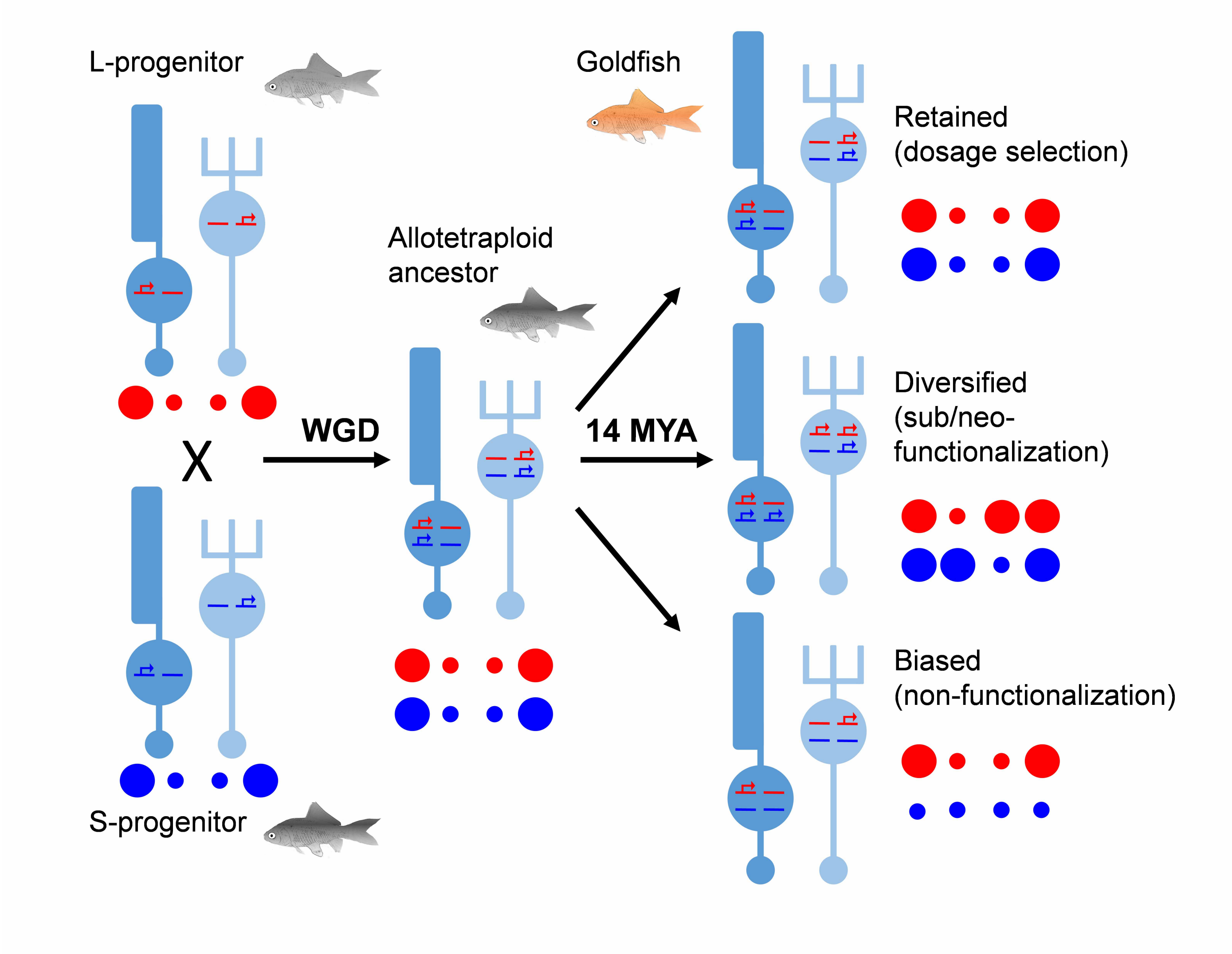

Supplementary Figure 11. Diagram showing the transcriptional divergence in the asymmetrically evolved subgenomes

The ancestor of goldfish experienced an allotetraploidization event 14 MYA. Ohnologs have various types of fates after WGD. A group of genes underwent dosage selection, accounting for 5% of the total 11,444 ohnolog pairs. In the goldfish retina, 306 putative sub/neo-functionalized ohnolog pairs were undergo cell-type-specific genetic variation. These diversifications of ohnolog expression patterns are likely to contribute to the functional evolution of the goldfish retina.
