# Supplementary material for: Single-cell transcriptomics of the goldfish retina reveals genetic divergence in the asymmetrically evolved subgenomes after allotetraploidization
Source: Commun Biol. 2022 Dec 26;5:1404. doi: 10.1038/s42003-022-04351-3 (PMC9792465; doi:10.1038/s42003-022-04351-3)
Supplement: Supplementary file 3 — Description of Additional Supplementary Files [file 42003_2022_4351_MOESM3_ESM.pdf]

Description of Additional Supplementary Files

File name: Supplementary Data 1
Description: Identified goldfish ohnolog pairs and corresponding zebrafish genes (11,444 sets).

File name: Supplementary Data 2
Description: Ohnolog pairs with cell-type-specific expression in which the L-ohnologs showed higher gene expression than the S-ohnologs.

File name: Supplementary Data 3
Description: Ohnolog pairs with cell-type-specific expression in which the S-ohnologs showed higher gene expression than the L-ohnologs.

File name: Supplementary Data 4
Description: Ohnolog pairs showing higher gene expression in the L-ohnolog than in the S-ohnolog (2,430 genes).

File name: Supplementary Data 5
Description: Ohnolog pairs showing higher gene expression in the S-ohnolog than in the L-ohnolog (2,188 genes).

File name: Supplementary Data 6
Description: Functional enrichment analysis of ohnolog pairs showing higher gene expression in the L-ohnolog than in the S-ohnolog (112 groups).

File name: Supplementary Data 7
Description: Functional enrichment analysis of ohnolog pairs showing higher gene expression in the S-ohnolog than in the L-ohnolog (129 groups).

File name: Supplementary Data 8
Description: Ohnolog pairs showing no significant difference in gene expression between the L-ohnolog and the S-ohnolog in any cell type (dosage-balanced, 611 genes).

File name: Supplementary Data 9
Description: Ohnolog pairs with different gene expression between the L-ohnolog and the S-ohnolog only in one cell type (762 genes).

File name: Supplementary Data 10
Description: Functional enrichment analysis of ohnolog pairs with different gene expression between the L- and S-ohnologs only in one cell type (295 groups).

File name: Supplementary Data 11
Description: Ohnolog pairs in which both the L- and S-ohnologs show cell-type-specific expression pattern (632 ohnolog pairs).

File name: Supplementary Data 12
Description: Functional enrichment analysis of the 326 ohnolog pairs in which both the L- and S-ohnologs exhibited the similar expression patterns (193 groups).

File name: Supplementary Data 13
Description: Functional enrichment analysis of the 306 ohnolog pairs in which both the L- and S-ohnologs exhibited different expression patterns (231 groups).

File name: Supplementary Data 14
Description: Open chromatin regions across the goldfish genome (245,817 sites).

File name: Supplementary Data 15
Description: Candidate regulatory regions of bias-expressed genes (L-bias 2,690 ohnolog pairs, 6,083 OCRs) based on scATAC-seq analysis.

File name: Supplementary Data 16
Description: Candidate regulatory regions of bias-expressed genes (S-bias 2,443 ohnolog pairs, 5,810 OCRs) based on scATAC-seq analysis.

File name: Supplementary Data 17
Description: Candidate regulatory regions of sub/neo-functionalization genes (306 ohnolog pairs, 849 OCRs) based on scATAC-seq analysis.

File name: Supplementary Data 18
Description: OCRs containing Otx2/Crx binding sites for regulation of the ohnolog pairs with photoreceptor/bipolar cell-specific expression.
