# Supplementary material for: Single-cell transcriptomics of the goldfish retina reveals genetic divergence in the asymmetrically evolved subgenomes after allotetraploidization
Source: Commun Biol. 2022 Dec 26;5:1404. doi: 10.1038/s42003-022-04351-3 (PMC9792465; doi:10.1038/s42003-022-04351-3)
Supplement: Supplementary file 5 — Reporting Summary [file 42003_2022_4351_MOESM5_ESM.pdf]

nature research

Corresponding author(s): Yoshihiro Omori

Last updated by author(s): 2022 Nov 9

Reporting Summary

Nature Research wishes to improve the reproducibility of the work that we publish. This form provides structure for consistency and transparency in reporting. For further information on Nature Research policies, see our Editorial Policies and the Editorial Policy Checklist.

Statistics

For all statistical analyses, confirm that the following items are present in the figure legend, table legend, main text, or Methods section.

n/a	Confirmed	
☐	☒	The exact sample size (n) for each experimental group/condition, given as a discrete number and unit of measurement
☐	☒	A statement on whether measurements were taken from distinct samples or whether the same sample was measured repeatedly
☐	☒	The statistical test(s) used AND whether they are one- or two-sided<br>*Only common tests should be described solely by name; describe more complex techniques in the Methods section.*
☐	☒	A description of all covariates tested
☐	☒	A description of any assumptions or corrections, such as tests of normality and adjustment for multiple comparisons
☐	☒	A full description of the statistical parameters including central tendency (e.g. means) or other basic estimates (e.g. regression coefficient) AND variation (e.g. standard deviation) or associated estimates of uncertainty (e.g. confidence intervals)
☐	☒	For null hypothesis testing, the test statistic (e.g. F, t, r) with confidence intervals, effect sizes, degrees of freedom and P value noted<br>*Give P values as exact values whenever suitable.*
☒	☐	For Bayesian analysis, information on the choice of priors and Markov chain Monte Carlo settings
☒	☐	For hierarchical and complex designs, identification of the appropriate level for tests and full reporting of outcomes
☒	☐	Estimates of effect sizes (e.g. Cohen's d, Pearson's r), indicating how they were calculated

Our web collection on statistics for biologists contains articles on many of the points above.

Software and code

Policy information about availability of computer code

Data collection	Hiseq 2500 (Illumina).
Data analysis	Seurat v4, cellranger, Metascape

For manuscripts utilizing custom algorithms or software that are central to the research but not yet described in published literature, software must be made available to editors and reviewers. We strongly encourage code deposition in a community repository (e.g. GitHub). See the Nature Research guidelines for submitting code & software for further information.

Data

Policy information about availability of data

All manuscripts must include a data availability statement. This statement should provide the following information, where applicable:

- Accession codes, unique identifiers, or web links for publicly available datasets
- A list of figures that have associated raw data
- A description of any restrictions on data availability

The NGS seq dataset in this studywas deposited in the DDBJ Sequence Read Archive (DRA) under accession numbers PRJDB12920.
Raw data for all the figures are available from the corresponding author upon reasonable request.

Field-specific reporting

Please select the one below that is the best fit for your research. If you are not sure, read the appropriate sections before making your selection.

☒ Life sciences ☐ Behavioural & social sciences ☐ Ecological, evolutionary & environmental sciences

For a reference copy of the document with all sections, see nature.com/documents/nr-reporting-summary-flat.pdf

Life sciences study design

All studies must disclose on these points even when the disclosure is negative.

Sample size	We described the number of animals and samples for all experiments in the text.<br>No statistical methods were used to predetermine sample size. The sample sizes were chosen based on published studies.
Data exclusions	No samples were excluded from the analysis.
Replication	Experiments were repeated in at least two independent preparations, and confirmed reproducibility of the results.<br>Individual numbers of replication are described in the text.
Randomization	Randomization was not employed.
Blinding	Data collection and analyses were not performed blind to the experimental condition.

Reporting for specific materials, systems and methods

We require information from authors about some types of materials, experimental systems and methods used in many studies. Here, indicate whether each material, system or method listed is relevant to your study. If you are not sure if a list item applies to your research, read the appropriate section before selecting a response.

Materials & experimental systems

n/a	Involved in the study	
☒	☐	Antibodies
☒	☐	Eukaryotic cell lines
☒	☐	Palaeontology and archaeology
☐	☒	Animals and other organisms
☒	☐	Human research participants
☒	☐	Clinical data
☒	☐	Dual use research of concern

Methods

n/a	Involved in the study	
☒	☐	ChIP-seq
☒	☐	Flow cytometry
☒	☐	MRI-based neuroimaging

Animals and other organisms

Policy information about studies involving animals; ARRIVE guidelines recommended for reporting animal research

Laboratory animals	Goldfish, Wakin. Goldfish was originally obtained from Japanese breeders. Wakin goldfish were maintained in Nagahama Institute of Bio-Science and Technology.
Wild animals	This study did not involve samples from wild animals.
Field-collected samples	This study did not involve samples collected from the fields.
Ethics oversight	All procedures were approved by the Institutional Safety Committee on Recombinant DNA Experiments (approval ID 0849) and the Animal Experimental Committees (approval ID 087) at Nagahama Institute of Bio-Science and Technology and were performed in compliance with institutional guidelines.

Note that full information on the approval of the study protocol must also be provided in the manuscript.
